# Spalling Resistance of Fiber-Reinforced Ultra-High-Strength Concrete Subjected to the ISO-834 Standard Fire Curve: Effects of Thermal Strain and Water Vapor Pressure

**DOI:** 10.3390/ma13173792

**Published:** 2020-08-27

**Authors:** Taegyu Lee, Gyuyong Kim, Gyeongcheol Choe, Euichul Hwang, Jaesung Lee, Dongwoo Ryu, Jeongsoo Nam

**Affiliations:** 1 Department of Fire and Disaster Prevention, Semyung University, 65 Semyung-ro, Jecheon-si, Choongbuk 27136, Korea; ninga777@naver.com; 2 Department of Architectural Engineering, Chungnam National University, Daejeon 34134, Korea; speed1382@nate.com (G.C.); sksdmlcjf@naver.com (E.H.); j.nam@cnu.ac.kr (J.N.); 3 Department of Architectural Engineering, Hannam University, 70 Hannamro, Daeduk-Gu, Daejeon 306-791, Korea; jaesung@hnu.kr; 4 Department of Architecture Engineering, Daejin University, Gyeonggi-do 11159, Korea; dwryu@daejin.ac.kr

**Keywords:** ultra-high-strength concrete, thermal stress, water vapor pressure, ISO-834 standard fire curve, pore formation, fiber melting, cross-sectional loss prevention

## Abstract

The prevention and mitigation of spalling in high-strength concrete (HSC) rely on mixing polypropylene (PP) as an additive reinforcement. The dense internal structures of ultra-high-strength concrete (UHSC) result in risks associated with a high thermal stress and high water vapor pressure. Herein, the effects of pore formation and thermal strain on spalling are examined by subjecting fiber-laden UHSC to conditions similar to those under which the ISO-834 standard fire curve was obtained. Evaluation of the initial melting properties of the fibers based on thermogravimetric analysis (TGA) and differential thermal analysis (DTA) demon strated that although nylon fibers exhibit a higher melting point than polypropylene and polyethylene fibers, weight loss occurs below 200 °C. Nylon fibers were effective at reducing spalling in UHSC compared to polypropylene and polyethylene fibers as they rapidly melt, leading to pore formation. We anticipate that these results will serve as references for future studies on the prevention of spalling in fiber-reinforced UHSC.

## 1. Introduction

Concrete is a composite material produced by mixing cement, an admixture, water, fine aggregates, and coarse aggregates. The structural matrix is solidified and hardened by a hydration reaction with the binder material (cement) [1]. High-strength concrete (HSC) with a low water/binder ratio is frequently used as a core construction material for high-rise structures due to its dense matrix structure. Moreover, the development and properties of ultra-high-strength concrete (UHSC) have also received growing interest in recent years [2,3,4,5].

Since HSC has a dense internal structure, there is a high risk of spalling, i.e., flaking of the surface layers, depending on the exposure time to fire. Spalling may cause building collapse due to a sudden decrease in the strength of the steel reinforcements when a cross-sectional loss of concrete occurs, as this results in a rapid temperature transference to the internal structure [5,6,7,8,9,10].

**Figure 1:**
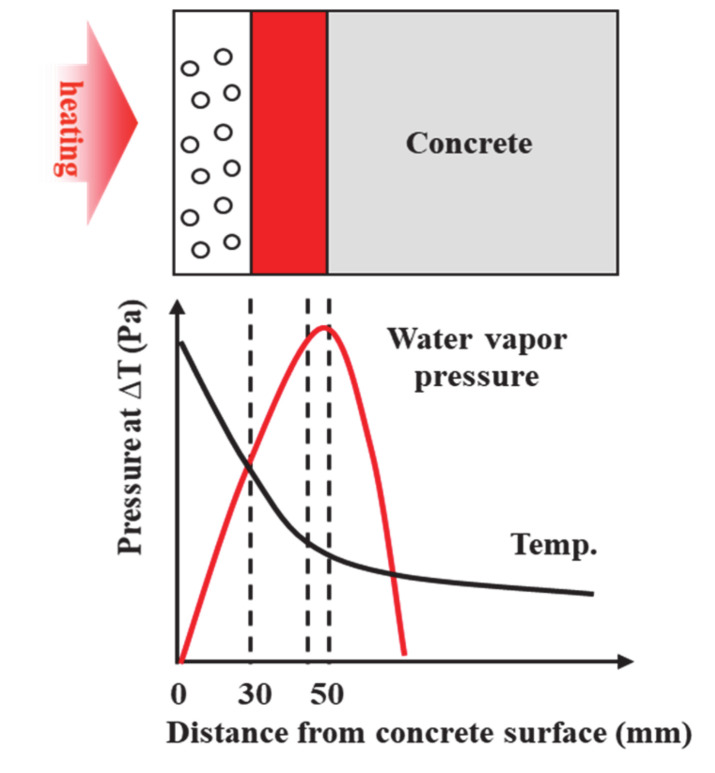
Change of water vapor pressure in concrete with temperature.

To date, the root causes of spalling in HSC have been extensively studied. The mechanism of spalling has been attributed to changes in the internal pressure due to moisture movement inside the concrete [11,12,13]. It has also been proposed that the concrete matrix collapses under the thermal stress caused by the thermal characteristics of various materials [14,15,16]. A dominant theory on the spalling of HSC is that spalling occurs when the tensile stress limit is exceeded due to internal moisture movement and water vapor pressure saturation caused by the elevated temperature, as seen in Figure 1.

**Figure 2:**
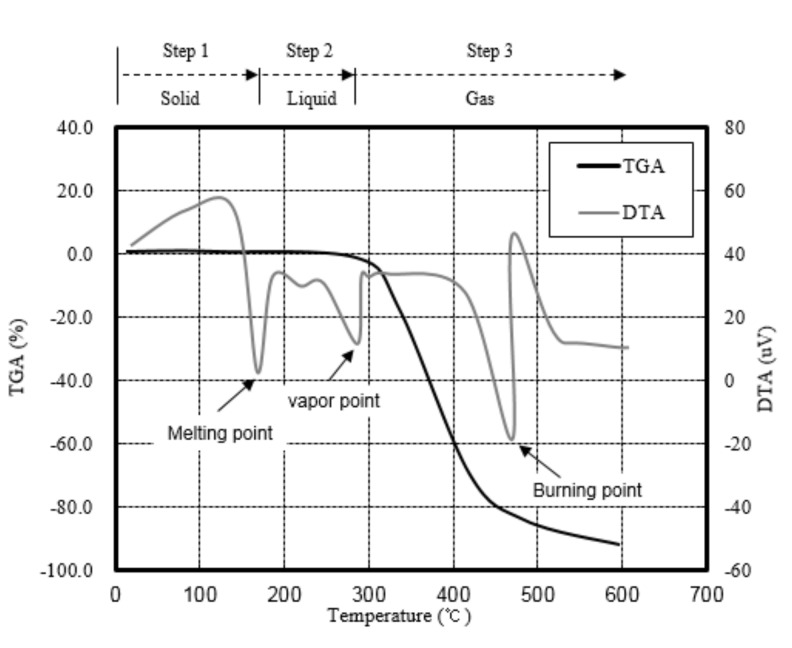
TGA (Thermogravimetric analysis) and DTA (Differential thermal analysis) profiles for various fibers, including the melting point, vapor point, and burning point.

Various methods have been proposed to prevent spalling in HSC, including reduction of the water vapor pressure of concrete by mixing fibers [17,18,19,20,21,22,23,24,25]. Indeed, this method has been established as a design standard in some countries [26]. The fibers used to prevent spalling in HSC experience rapid phase changes from solids to liquids and gases, as shown in Figure 2.

Fibers with low melting points have been reported to be favorable for reducing the internal water vapor pressure of the resulting HSC [16,18,23,24]. More specifically, Kalifa et al. [18] reported that the spalling of UHSC with a 100 MPa compressive strength could be prevented by mixing the concrete with approximately 2.0 kg/m^3^ of polypropylene (PP) fiber, and found that pore networks were formed over melting point of fiber due to the generation of microcracks adjacent to the added fiber. However, the incorporation of these fibers did not prevent spalling between 80–200 °C.

It is also known that the internal matrix structures within UHSC are more brittle than those of HSC due to the presence of binders such as silica fume and gypsum, in addition to superplasticizers. UHSC may also be vulnerable to spalling due to the small number of internal pores within its structure. It is, therefore, necessary to reduce the internal water vapor pressure of concrete by adding specifically selected fibers for anticipated fire conditions and to fully examine the influence of thermal stresses that occur during the heating process [27,28,29].

Thus, we herein wish to examine the relationship between spalling and the melting characteristics of additive fibers under scenarios representative of the ISO-834 standard fire curve by mixing UHSC with polyethylene (PE), PP, and nylon (NY) fibers with various melting points. We examined the effects of these different additive fibers and their concentrations (0, 0.15, and 0.25% of the concrete volume) when applied to UHSC. The observed evaluation metrics included the grade according to the spalling property, thermal strain, and water vapor pressure.

## 2. Materials and Methods

### 2.1. Materials

Table 1 and Table 2 list the physical properties of the materials employed in this study, whereby Type 1 ordinary Portland cement (density: 3150 kg/m^3^, fineness: 330 m^2^/kg, Hanil Cement, Seoul, Korea) was used.

**Figure 3:**
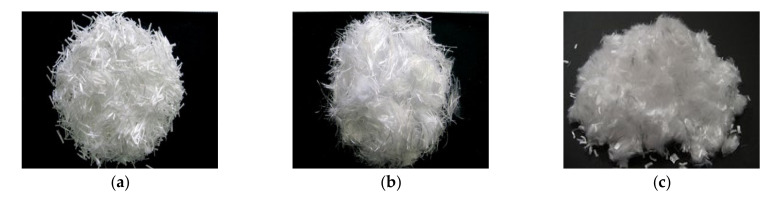
Fiber type: (**a**) Polyethylene (PE); (**b**) polypropylene (PP); (**c**) nylon (NY).

For the mineral admixtures, ground granulated blast-furnace slag (density: 2860 kg/m^3^, fineness: 430 m^2^/kg, Hanil Cement, Seoul, Korea), silica fume (density: 2500 kg/m^3^, fineness: 20,000 m^2^/kg, Elkem, Oslo, Norway), and gypsum (density: 2900 kg/m^3^, fineness: 355 m^2^/kg, Yusung-tech, Daejeon, Korea) were used. Sea sand (FM (Fineness modulus): 2.90, density: 2650 kg/m^3^, absorption: 1.00%, Asung Industry Development, Chungcheongnam-do, Korea) was used as the fine aggregate, and crushed granitic aggregate (size: 13 mm, density: 2700 kg/m^3^, absorption: 0.90%, Asia Industry Development, Sejong, Korea) was used as the coarse aggregate. PE, PP, and NY fibers were used to evaluate their effects on concrete spalling. Figure 3 shows the three fiber types.

### 2.2. Experimental Plan and Concrete Mixture Proportions

Table 3 presents the experimental plan employed in this study.

The compressive strength of the concrete was set at 150 MPa. To analyze the correlations between the fiber characteristics and concrete spalling, PE, PP, and NY were specifically selected as additive fibers for mixing with the concrete, considering the fiber melting point and diameter. The fiber concentrations were set to 0, 0.15, and 0.25% based on the concrete volume [11].

The concrete specimens were heated for 50 min under conditions representative of the ISO-834 standard fire curve [30]. The spalling properties, thermal strain, weight losses, and water vapor pressures of the concrete specimens were evaluated. Table 4 lists the mixture proportions of the concrete.

The fibers were mixed with UHSC using a mixing machine (double axial spiral mixing type, mixing capacity: 180 L, mixing speed: 5–50 rpm, Woojin, Korea). Silica fume and coarse aggregate were then added. After mixing for 30 s, the fine aggregate was added to the mixture and mixed for a further 30 s, and then the binder, fiber, and water were each added and mixed for 30 s. In order to ensure sufficient fluidity, a superplasticizer was added and mixed for 300 s. After confirming the fluidity of the concrete, further mixing was performed for 50 s. It took a total of 440 s to create the fiber-blended UHSC.

The water/binder ratio was set to 0.13 to represent the compressive strength of concrete, where the unit water content was 150 kg/m^3^. The slump flow [31] was set to 750 ± 100 mm to ensure sufficient workability, and the air content [32] was maintained at 2 ± 1%. The target concrete parameters under fiber reinforcement were met at all levels. The concrete specimens evaluated for spalling characteristics were fabricated in a hexahedron form with 100-mm width, 100-mm depth, and 200-mm height. The specimens were cured under air for 56 days in a constant temperature and humidity chamber at 20 ± 2 °C and 60 ± 5%, respectively [33].

### 2.3. Test Methods

#### 2.3.1. Heating Method

**Figure 4:**
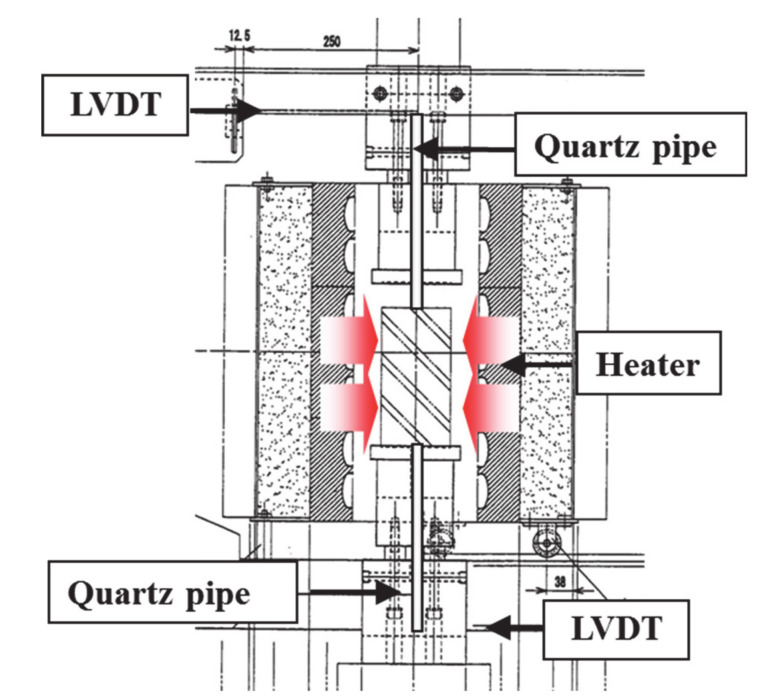
Schematic representation of the heating method.

**Figure 5:**
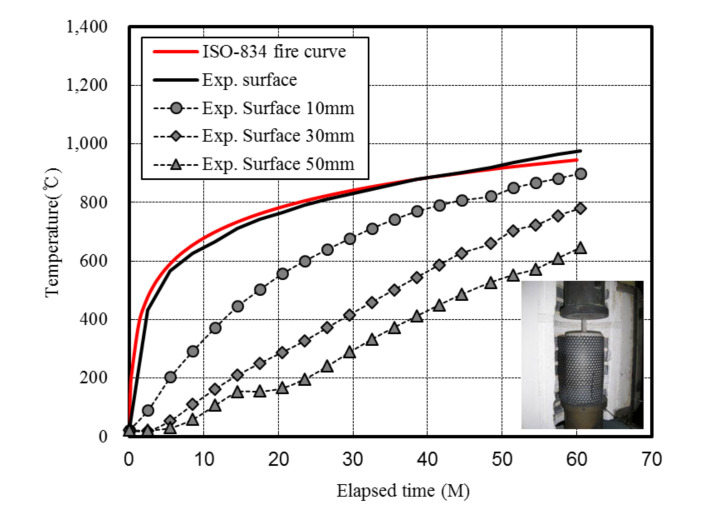
ISO-834 fire curve and inner temperature histories of the concrete specimen.

Figure 4 and Figure 5 demonstrate the heating method and heating curve employed herein.

Within our experimental setup, an electric furnace was installed in a 2000 kN class universal testing machine (UTM, SHIMADZU, Kyoto, Japan) using a steel frame. The temperature was adjusted by applying heat to replicate the conditions of the ISO-834 standard fire curve through preliminary experiments and literature results [34,35,36,37]. Adjustments were implemented by modulating the electric heater temperature based on measurement of the specimen temperature using a thermocouple (Ø1.6 × 3000 mm sheath + 0.65 mm^2^ × 1000 mm thermo shield wire, Jooshin, Incheon, Korea). Based on the prescribed scenario and measurements, the relationships between time and temperature were obtained by varying the temperature.

Each specimen was heated for 50 min based on the ISO-834 standard fire curve. Observations during a preliminary simulation on concrete revealed that the surface temperature increased following the set ISO-834 standard fire curve.

#### 2.3.2. Thermal Strain and Weight Loss

**Figure 6:**
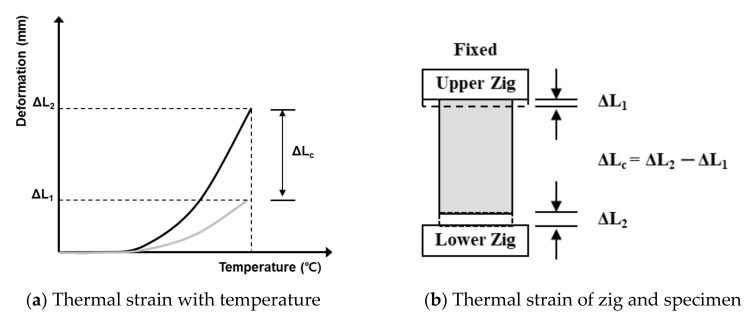
Test method for determining the thermal strain.

Figure 6 displays the thermal strain evaluation method.

Quartz pipes were installed in the upper and lower loading plates and at the center of the jig; ∅15-mm space and strain gauges were installed at both ends of the concrete specimen to measure the thermal strain (see Figure 3). The thermal strain of the specimen was calculated using Equation (1), based on the observed expansion of the jigs and specimen:

(1) $$\Delta L_{C}=\Delta L_{2}-\Delta L_{1},$$

where Δ*L*_c_ is the thermal strain of concrete, Δ*L*_2_ is the strain of the lower strain gauge, and Δ*L*_1_ is the strain of the upper strain gauge.

The weight loss of the concrete specimen was calculated using Equation (2) by measuring the weight of the specimen before and after heating using an electronic scale with 1/100 precision [23,24]:

(2) $$\Delta W_{C}\left(\%\right)=\;\frac{\left(W_{\mathrm{after}}-W_{\mathrm{initial}}\right)}{W_{\mathrm{initial}}},$$

where Δ*W*_c_ is the weight loss of concrete, *W*_after_ is the weight of concrete after heating, and *W*_initial_ is the weight of concrete before heating. The weight loss of concrete due to spalling during heating was measured to evaluate and quantify the spalling properties. The spalling grade based on the weight loss of concrete was defined as follows:

- Major spalling: Weight loss percentage > 1/3.
- Minor spalling: Weight loss percentage < 1/3.
- No spalling: No or slight spalling.

In addition, spalling was recorded at the start and end points of the experiment when large amounts of water were present.

#### 2.3.3. Water Vapor Pressure

**Figure 7:**
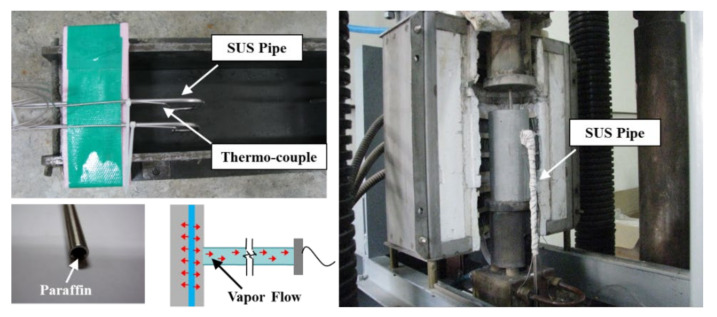
Test method for determining the water vapor pressure.

Figure 7 displays the test method used to determine the water vapor pressure inside the concrete.

The water vapor pressure measurement method used in this study was measured by releasing water vapor directly, not the indirect measurement method published in previous studies [16,17,18]. The test device was configured in consideration of the release of water vapor pressure and the temperature of water vapor during measurement through sufficient simulation tests in advance.

A cylindrical stainless steel (SUS No. 303, Woojin, Gyeonggi-do, Korea) pipe (*Ø*6 mm) that displayed an excellent temperature resistance was used as a pressure pipe for measuring the water vapor pressure in the concrete specimens. The pressure pipe inlet was completed with a low melting point paraffin to prevent the inflow of aggregates and pastes during fabrication of the concrete specimens. The pressure pipe was pre-installed inside a mold measuring 100 mm × 100 mm × 200 mm, into which the concrete was poured. Two test specimens for pressure measurements were prepared for each concrete mixture.

The pressure pipes were installed at approximately 30 and 50 mm, respectively, from the surface of the concrete specimen based on the results of previous studies [8,11,13]. To measure the water vapor pressure, the buried stainless steel pipe and the tip of a pressure sensor were fastened via a coupler, preventing pressure leakage, to generate an instrument capable of carrying out pressure measurements. A pressure gauge (PHF-S-SA2, Tokyo Sokki Kenkyujo Co., Tokyo, Japan) capable of performing at 150 °C was used as the pressure sensor to obtain the water vapor temperature. The pressure measurement capacity of this apparatus was 20 MPa.

## 3. Results and Discussion

### 3.1. Spalling Grade Based on the Weight Loss of Concrete

**Figure 8:**
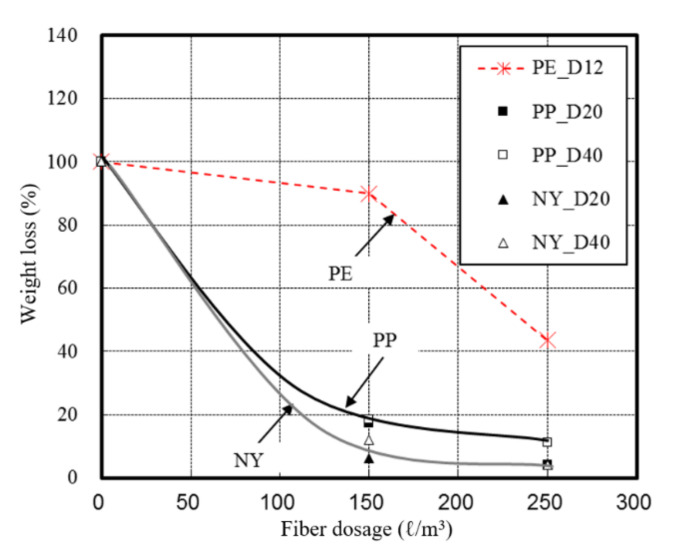
Weight loss of concrete where spalling occurs for different fibers.

Figure 8 illustrates the weight loss of concrete with respect to the fiber dose.

**Figure 9:**
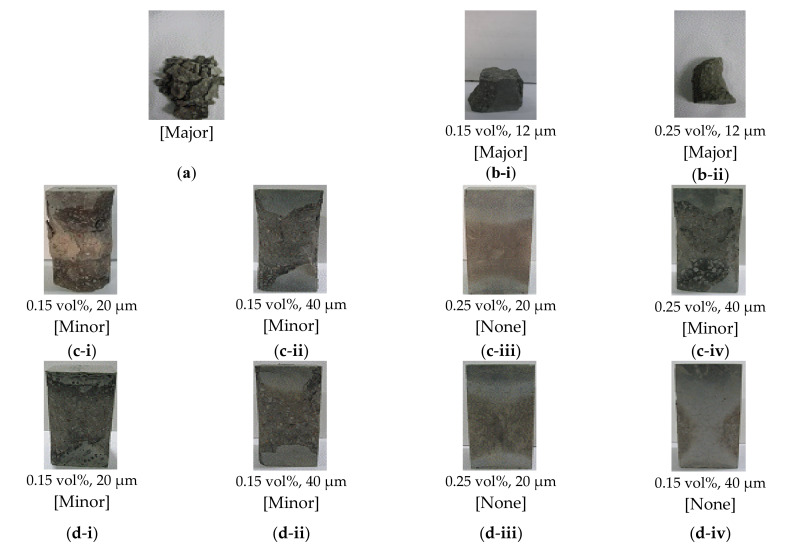
Spalling grades and images of the concrete specimens with additive fibers: (**a**) Plain; (**b**) polyethylene (PE); (**c**) polypropylene (PP); (**d**) nylon (NY).

The spalling grade was classified according to the weight loss of the concrete, based on the previously discussed scale. Figure 9 shows the spalling grades of the specimens, according to their respective weight losses.

Examination of the weight loss respective to the fiber content indicated that the weight loss of the UHSC specimens decreased as the fiber content increased. Similar to previous studies [11,14], this observation was attributed to a reduction in the cross-sectional loss of concrete caused by spalling. Fibers with smaller diameters were observed to provide more favorable spalling results.

Evaluation of the spalling properties indicated that spalling generally occurred when the fiber concentration was 0.15 vol%. However, both plain and PE fiber-reinforced specimens exhibited major spalling, while the incorporation of PP and NY fibers resulted in only minor spalling. With 0.25 vol% fiber content, the specimens that contained PE fibers also exhibited major spalling. The PP fibers, however, only resulted in minor spalling, with only surface scaling observed. The specimens with NY fibers did not display spalling.

Among the various additive fiber materials examined in this study, the PE fibers exhibited the lowest melting point (157 °C), followed by the PP fibers (167 °C), and the NY fibers (220 °C). However, the PE fibers resulted in the most severe UHSC spalling, followed by PP and NY fibers. These findings contrasted those of previous studies that reported that fibers with low melting points favorably prevented spalling in HSC. These conflicting results indicate that a more detailed analysis of the spalling mechanism in UHSC is required.

### 3.2. Thermal Strain with Spalling Properties

**Figure 10:**
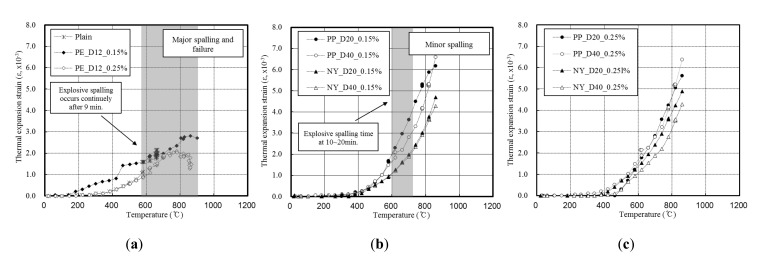
Thermal strain of the fiber-mixed concrete specimens with: (**a**) Major spalling; (**b**) minor spalling; (**c**) no spalling.

Figure 10 shows the thermal strain of the concrete specimens, according to their spalling properties.

For the plain and PE specimens, which exhibited major spalling, the thermal strain increased slowly with temperature (Figure 10a). In addition, thermal expansion decreased during the onset of spalling, typically between 7 and 10 min, while the deformation of the concrete increased continuously as spalling took place upon increasing the temperature. However, it should also be noted that at 800 °C, the thermal strain decreased to 2.0 × 10^−3^ due to severe cross-sectional loss.

For the concrete specimens mixed with PP and NY, which displayed only minor or no spalling, it was observed that the thermal strain continuously increased as the temperature increased (Figure 10b,c). Furthermore, the thermal strain in the concrete increased as the fiber diameter decreased, and the concrete mixed with NY fibers exhibited lower thermal strain than that mixed with PP fibers.

### 3.3. Water Vapor Pressure of Concrete with Spalling Properties

**Figure 11:**
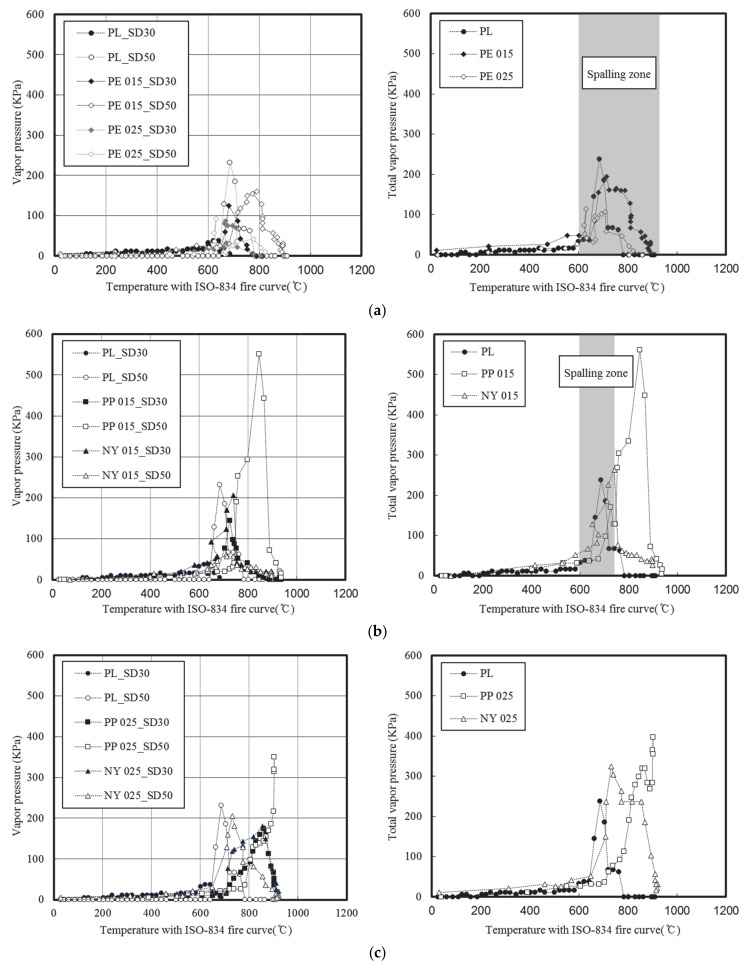
Water vapor pressures of the fiber-mixed concrete specimens with: (**a**) Major spalling; (**b**) minor spalling; (**c**) no spalling.

Figure 11 presents the water vapor pressures of the concrete specimens according to their degree of spalling.

The maximum water vapor pressure was reached between 10 and 20 min, coinciding with the onset of spalling, while the pressure maximum was reached at a distance of 50 mm from the surface between 20 and 30 min, i.e., at a point where spalling was suppressed by high concentrations of the additive fiber. In addition, major spalling occurred in both the plain and PE fiber-modified specimens. Spalling occurred after 6 min of heating at 637 °C for the plain specimens; the pressure 30 mm from the surface was 43.2 kPa during the initial spalling.

Measuring the water vapor pressure in both the plain and PE was challenging because the continuous spalling caused sudden cross-sectional losses. Furthermore, at a distance of 50 mm from the surface, the water vapor pressure rapidly increased after 15 min due to the onset of spalling; the specimens finally collapsed as their strength rapidly decreased after the maximum pressure of 231.8 kPa was reached at 21 min during heating at 685 °C. Upon the incorporation of PE fibers, a water vapor pressure 30 mm from the surface was formed simultaneously with the onset of spalling at a fiber concentration of 0.15 vol%; a maximum pressure of 124.2 kPa was observed at ~11 min. At a distance of 50 mm from the surface, a maximum pressure of 157 kPa was observed at 20 min. When the fiber content was 0.25 vol%, a similar behavior to the plain concrete was initially observed. However, observations confirmed that the water vapor pressure at a depth of 30 mm was reduced.

Minor spalling occurred in the concrete specimens mixed with either 0.15 vol% PP or NY. The maximum water vapor pressure at a distance of 30 mm from the surface formed between 10 and 20 min, with values of 144.2 and 206 kPa recorded for the specimens mixed with PP and NY fibers, respectively. Furthermore, at a distance of 50 mm from the surface, the specimen mixed with NY fibers maintained an approximate pressure of 61.8 kPa, while the PP-laden specimen exhibited a pressure of 551 kPa.

No spalling occurred in the specimens mixed with 0.25 vol% PP and NY. In these cases, water vapor pressure was not measured until 15 min of heating occurred. The specimens mixed with either PP or NY fibers exhibited similar water vapor pressure patterns at a distance of 30 mm from the surface, and the specimen mixed with NY fibers exhibited higher water vapor pressures at an earlier point in the experiment. At a distance of 50 mm from the surface, the specimens mixed with either PP or NY fibers exhibited drastically different water vapor pressure behaviors. More specifically, for the specimen mixed with NY fibers, the water vapor pressure increased until 12–18 min, then slowly decreased after reaching 206 kPa. The PP fiber specimen did not display this phenomenon until the 20-min mark. Subsequently, the pressure slowly increased, reaching 350 kPa. All the UHSC mixtures displayed similar values (15–30 kPa) during the onset of spalling, yet the specimen mixed with PP fibers exhibited a higher pressure at 50 mm. As the fiber concentration was reduced, higher degrees of spalling were observed with increasing temperatures, due to surface scaling of the concrete.

### 3.4. Relationship between the Thermal Strain and the Water Vapor Pressure

**Figure 12:**
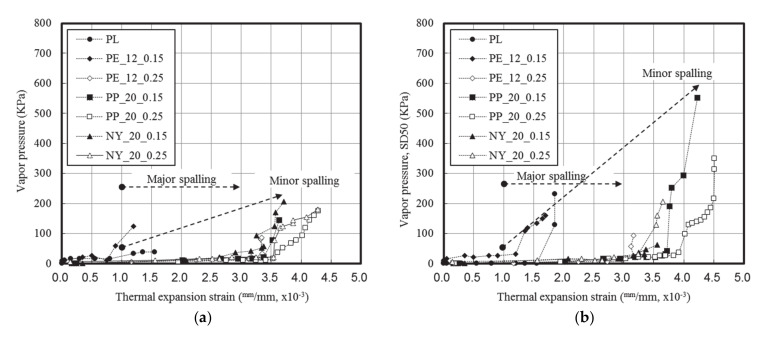

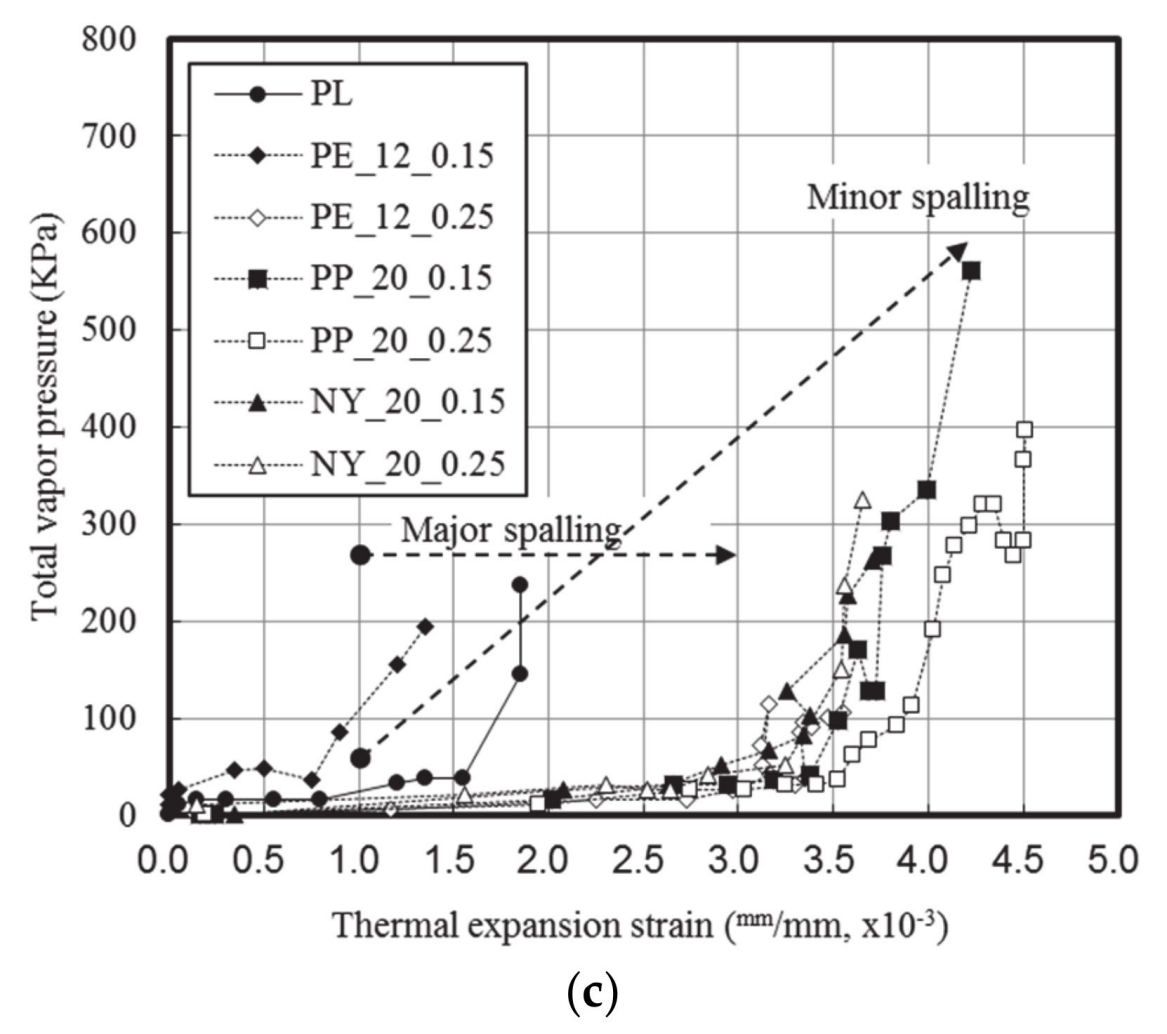
Relationship between thermal strain and water vapor pressure: (**a**) Distance from surface, 30 mm; (**b**) distance from surface, 50 mm; (**c**) accumulated amount.

Figure 12 displays the relationship between the thermal strain and the water vapor pressure.

For the fiberless and PE fiber-reinforced UHSC specimens, which typically displayed major spalling, spalling occurred continuously within the thermal strain range of 1.0 × 10^−3^ to 2.0 × 10^−3^. Following a rapid increase in the water vapor pressure, the subsequent decrease was attributed to the influence of cross-sectional loss.

In the case of the UHSC specimens mixed with either 0.15 vol% PP or NY fibers, minor spalling began at a thermal strain of 1.0 × 10^−3^. Both specimens exhibited similar water vapor pressure values at 30 mm, but the specimen that contained PP fibers displayed a higher thermal strain and water vapor pressure than that mixed with NY fibers.

In the case of the UHSC specimens mixed with either 0.25 vol% PP or NY fibers, no spalling was observed, and the water vapor pressure decreased slightly compared to the specimens mixed with 0.15 vol% fibers. The specimen mixed with PP fibers exhibited a 1.0 × 10^−3^ higher thermal strain than the specimen mixed with NY fibers.

**Figure 13:**
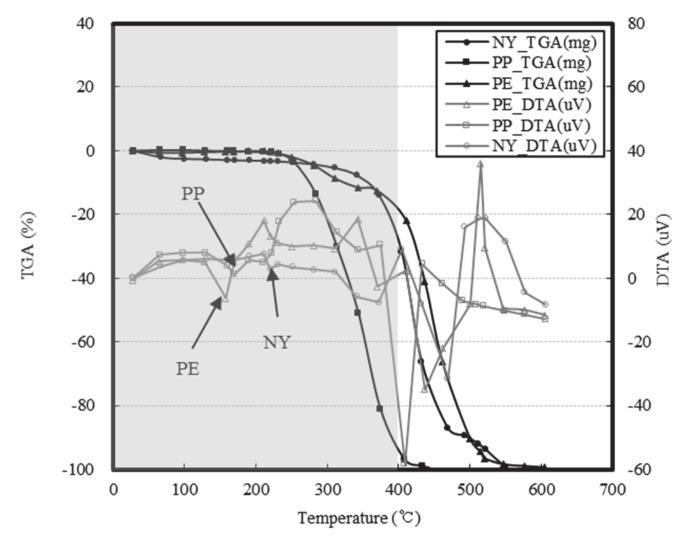
Thermal properties of the fibers employed in this study including the TGA and DTA results.

Upon heating of the concrete, a high water vapor pressure was obtained at a distance of 30 mm from the surface. We note that spalling can be prevented by dispersing the water vapor throughout the materials along the additive fibers. In the case of UHSC, the saturation point of the water vapor pressure was closer to the surface due to the denser matrix structure; additionally, our observations indicated that the addition of fibers can shift the maximum vapor pressure point within the concrete. Indeed, Phan [13] and Kalifa et al. [18] reported that dispersion of the water vapor by additive fibers was an effective means of preventing spalling. However, in the case of UHSC, an increase in the fiber content increased the internal pressure; these effects were attributed to increased deformation and water vapor movement caused by thermal stress. Although previous studies have reported that fibers with lower melting points were more favorable for preventing spalling, these studies were unable to account for the (seemingly contradictory) finding that NY fibers resulted in better spalling prevention than either the PE or PP fibers in the temperature range shown in Figure 13.

**Figure 14:**
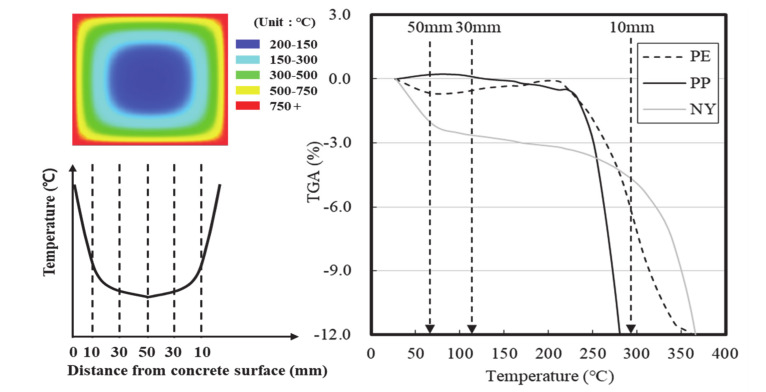
Initial melting properties of the fibers based on the TGA and DTA measurements.

Figure 14 displays the initial melting properties of the fibers by concrete depth, according to the heating temperature.

**Figure 15:**
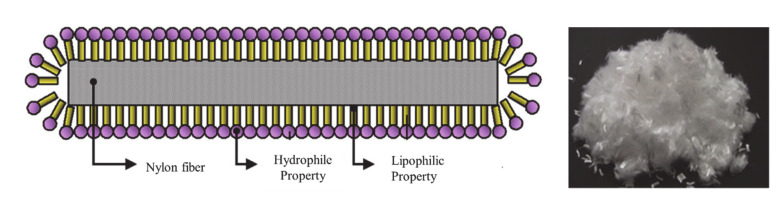
Surface coating of the nylon fiber.

The temperatures recorded during this analysis were the internal temperatures of the concrete specimens after 10 min of heating. Despite the PE and PP fibers exhibiting low melting points, they did not rapidly generate pore networks. Moreover, it was difficult to prevent spalling with PP fibers, even at high concentrations, because the PP fibers generally increased in weight between 100 and 300 °C, resulting in pore closure. Despite their high melting point, observations indicated that NY fibers were more effective at preventing spalling than the other fibers due to the rapid melting of ~3–4% of the fibers to generate pores between 100–400 °C. As shown in Figure 15, the NY fibers employed herein were coated with a dispersant consisting of 40–50% alcohol ester, 30–40% anionic surfactant, and 10–30% antistatic agent.

Therefore, when the concrete was subjected to high temperatures, the coating agent of the NY fibers rapidly evaporated, potentially resulting in the formation of fine pores.

## 4. Conclusions

We examined the effects of the thermal strain and the water vapor pressure with respect to the melting properties of fibers on UHSC specimens that displayed several grades of spalling. The spalling properties of several types of fiber-reinforced UHSCs were classified into major spalling, minor spalling, and no spalling, according to the observed weight loss. It was observed that PE and PP fibers with low melting points caused spalling, and minor spalling occurred even when the fiber concentration was increased. In addition, the thermal stress did not increase continuously due to spalling generating a cross-sectional loss in both the fiberless and PE fiber-reinforced specimens. The thermal strain continuously increased with temperature for the specimens mixed with either PP or NY fibers, and the thermal strain of concrete increased as the additive fiber diameter decreased. In general, the PP fibers demonstrated higher thermal strains than the NY fibers. It was determined that the thermal stress was large, even with reduced spalling. Furthermore, we found that the trapped moisture within the UHSC moved to the interior via pore networks generated by the melting fibers. Moreover, at a constant fiber concentration, the maximum water vapor pressure was measured under heating, and the reduction in water vapor pressure caused by the NY fibers was greater than those of the other fibers. In addition, we observed that the NY fibers, whose weight loss occurred at temperatures <200 °C, were more effective at preventing spalling than either the PE or PP fibers. This was likely due to the NY fibers reducing the water vapor pressure and thermal stress through pore formation upon evaporation of the surface modification coating. Overall, UHSC specimens with large concentrations of PP fibers displayed good resistance to spalling. However, we note that the effects of thermal stress and water vapor pressure on the spalling of fiber-reinforced UHSC were evaluated using only small specimens. It is, therefore, possible that these specimens may not be representative of general spalling properties, as both the thermal stress and moisture content can differ with scale. Nevertheless, we anticipate that the results of this study could serve as a reference for future studies seeking to prevent spalling in fiber-reinforced UHSC.

In the scope of this study, it was confirmed that not only the melting point of the fiber but also the surface treatment of the fiber influenced the spalling of fiber-reinforced UHSC. Therefore, in future studies, in-depth research on the surface treatment of fibers is required. In addition, additional studies should be conducted according to the characteristics of the fibers under the same surface treatment conditions.

## Figures and Tables

**Table 1:** Chemical compositions of the binders.

Material	Chemical Composition (wt.%)								
	CaO	SiO_2_	Al_2_O_3_	Fe_2_O_3_	MgO	K_2_O	Na_2_O	TiO_2_	SO_3_
OPC ^1^	60.20	21.60	5.15	3.30	2.30	0.99	0.53		1.50
GGBS ^2^	44.90	35.40	13.00	0.47	5.01	0.37			1.31
SF ^3^	0.16	95.30	0.06	0.02	0.30	0.31			
Gy ^4^	41.57	0.73	0.17	0.16		0.03			55.5

^1^ OPC: ordinary Portland cement; ^2^ GGBS: ground granulated blast-furnace slag; ^3^ SF: silica fume; ^4^ Gy: gypsum

**Table 2:** Physical properties of the materials.

Material		Property
OPC		ASTM Type I ordinary Portland cement Density: 3150 kg/m^3^, fineness: 330 m^2^/kg
GGBS		Ground granulated blast-furnace slag Density: 2860 kg/m^3^, fineness: 430 m^2^/kg
SF		Silica fume Density: 2500 kg/m^3^, fineness: 20,000 m^2^/kg
Gy		Gypsum Density: 2900 kg/m^3^, fineness: 355 m^2^/kg
Fine Aggregate		Washings sand Fineness modulus: 2.90, density: 2650 kg/m^3^, absorption: 1.00%
Coarse Aggregate		Crushed granitic aggregate Size: 13 mm, density: 2700 kg/m^3^, absorption: 0.90%
Fiber	PE	Polyethylene Diameter: 12 μm, length: 12 mm, density: 910 kg/m^3^, melting point: 110 ℃
	PP	Polypropylene Diameter: 20, 40 μm, length: 12 mm, density: 910 kg/m^3^, melting point: 165 ℃
	NY	Nylon Diameter: 20, 40 μm, length: 12 mm, density: 1100 kg/m^3^, melting point: 225 ℃
Super Plasticizer (SP)		polycarboxylate -based super plasticizer

**Table 3:** Experimental plan.

Water-Binder Ratio	Fiber Type (Diameter)	Fiber Dosage (% by Volume)	Heating (℃)	Test Item
0.13	Plain	0.0	ISO-834 Standard fire curve	▪ Spalling properties ▪ Thermal strain ▪ Weight loss (wt.%) ▪ Water vapor pressure (kPa)
	PE (12 μm)	0.15 0.25		
	PP (20, 40 μm)	0.15 0.25		
	NY (20, 40 μm)	0.15 0.25		

**Table 4:** Mixture proportions and properties of fresh and hardened concretes.

Concrete Type		Plain	PE	PP		NY	
			D12	D20	D40	D20	D40
Cement Content Type 1 (kg/m^3^)		660	660	660	660	660	660
Silica Fume (kg/m^3^)		240	240	240	240	240	240
GGBS ^1^ (kg/m^3^)		240	240	240	240	240	240
Gypsum (kg/m^3^)		60	60	60	60	60	60
Fine Aggregate (kg/m^3^)		389	389	389	389	389	389
Coarse Aggregate (kg/m^3^)		736	736	736	736	736	736
Water (kg/m^3^)		150	150	150	150	150	150
Water/Binder Ratio		0.13	0.13	0.13	0.13	0.13	0.13
Fiber contents (vol%)		0	0.15/0.25	0.15/0.25	0.15/0.25	0.15/0.25	0.15/0.25
Fresh concrete							
Slump-flow (mm)		785	700/580	780/760	790/760	800/760	800/720
Air contents (vol%)		2.1	2.0/3.1	2.5/2.8	2.7/3.0	2.0/2.3	2.4/2.2
Hardened Concrete							
Compressive strength (MPa)	28 days	173	163/154	171/159	175/168	161/164	170/173
	56 days	175	167/156	173/168	177/168	175/170	176/173

^1^ GGBS: ground granulated blast-furnace slag.
